# The Safety of Removing Fractured Nickel–Titanium Files in Root Canals Using a Nd: YAP Laser

**DOI:** 10.3390/biomedicines12051031

**Published:** 2024-05-07

**Authors:** Amaury Namour, Marwan El Mobadder, Patrick Matamba, Lucia Misoaga, Delphine Magnin, Praveen Arany, Samir Nammour

**Affiliations:** 1 Department of Dental Science, Faculty of Medicine, University of Liege, 4000 Liege, Belgium; amaurynamour@gmail.com (A.N.); marwan.mobader@gmail.com (M.E.M.); patrickmatamba@gmail.com (P.M.); luciamisoaga@gmail.com (L.M.); 2 Laser Laboratory, Oral Surgery Department, Wroclaw Medical University, 50-425 Wroclaw, Poland; 3 Bio- and Soft Matter Division, Institute of Condensed and Nanosciences, Université Catholique de Louvain (UCL), 1348 Louvain-la-Neuve, Belgium; delphine.magnin@uclouvain.be; 4 Oral Biology, Biomedical Engineering & Surgery, University at Buffalo, Buffalo, NY 14214, USA; prarany@buffalo.edu

**Keywords:** endodontic files, root canal treatment, root canal re-treatment, fractured instruments, nickel, titanium, endodontics, Nd: YAP laser, lasers, laser dentistry

## Abstract

The fracture of nickel–titanium (Ni-Ti) instruments during root canal instrumentation leads to compromised outcomes in endodontic treatments. Despite the significant impact of instrument facture during a root canal treatment, there is still no universally accepted method to address this complication. Several previous studies have shown the ability of a Neodymium: Yttrium–Aluminum–Perovskite (Nd: YAP) laser to cut endodontic files. This study aims to determine safe irradiation conditions for a clinical procedure involving the use of a Neodymium: Yttrium–Aluminum–Perovskite (Nd: YAP) laser for removing fractured nickel–titanium files in root canals. A total of 54 extracted permanent human teeth (*n* = 54) were used. This study involved nine distinct groups, each employing different irradiation conditions. Groups 1 s, 3 s, 5 s, 10 s, and 15 s simply consist of irradiation for 1, 3, 5, 10, and 15 s, respectively. After identifying the longest and safest duration time, four additional groups were proposed (labeled A, B, C, and D). Group A was composed of three series of irradiations of 5 s each separated by a rest time of 30 s (L5s + 30 s RT). Group B consisted of three series of irradiations of 5 s each separated by a rest time of 60 s (L5s + 60 s RT). Group C consisted of two series of irradiations of 5 s each separated by a rest time of 30 s (L5s + 30 s RT), and group D consisted of two series of irradiations of 5 s each separated by a rest time of 5 s (L5s + 5 s RT). In all groups, during the rest time, continuous irrigation with 2.5 mL of sodium hypochlorite (3% NaOCl) was carried out. The variation in temperature during irradiation was registered with a thermocouple during irradiation with different protocols. The mean and standard deviation of the temperature increase was noted. The calculation of the temperature was made as the Δ of the highest recorded temperature at the root surface minus (−) that recorded at baseline (37°). Additionally, scanning electron microscopy (SEM) was used after irradiation in all groups in order to assess the morphological changes in the root dentinal walls. The Nd: YAP laser irradiation parameters were a power of 3W, an energy of 300 mJ per pulse, a fiber diameter of 200 µm, a pulsed mode of irradiation with a frequency of 10 Hz, a pulse duration of 150 µs, and an energy density of 955.41 J/cm^2^. Our results show that the safest protocol for bypassing and/or removing broken instruments involves three series of irradiation of 5 s each with a rest time of 30 s between each series. Furthermore, our results suggest that continuous irradiation for 10 s or more may be harmful for periodontal tissue.

## 1. Introduction

In root canal treatment and re-treatment, numerous complications can occur during the cleaning and shaping of root canals. While some complications can be resolved, others can significantly reduce the prognosis and sometimes lead to complete treatment failure [1,2,3]. In this context, the fracture of instruments in the root canal (RC) during canal shaping is reported to be one of the most common reasons for a negative prognosis [4,5]. The introduction of nickel–titanium (Ni-Ti) instruments two decades ago revolutionized endodontic treatments [6] thanks to their versatile alloy with properties such as memory, super elasticity, corrosion resistance, resistance to torsional fracture, and biocompatibility [6]. However, like all endodontic instruments, Ni-Ti instruments can fracture [1,2,3]. The prevalence of retained fractured endodontic instruments is reported to be approximately 1.6% with a range of 0.7–7.4%; however, it should be noted that studies may not provide representative data since not all fractured instruments are documented [7,8,9,10]. To address this complication, numerous studies have introduced special instruments and techniques for retrieving obstructing objects, such as ultrasonic instruments [11], hollow tubes with a cyanoacrylate adhesive [11], trephining techniques using an ultrasonic tip or a trepan bur [12], endo-extractors [13], and welding with a Neodymium: Yttrium–Aluminum–Perovskite (Nd: YAG) laser [14]. Surgical techniques for the removal of either the instrument itself or the entire portion of the root encompassing the instrument have also been described [15,16,17,18,19]. For instance, Gencoglu et al. [11] obtained a success rate of 93.3% when ultrasonic tips were used under magnification compared to only a 66.6% success rate when conventional methods were used in curved canals [11]. Suter et al. [20] concluded in a study that 87% of fractured instruments can be removed successfully with proper protocols if operating under a microscope [20]. On the contrary, Barbara et al. [21] utilized a Nd: YAP laser in a specific protocol to remove in vitro broken instruments, achieving success rates of 77.3% and 27% depending on the scenario of the broken instrument [21]. In their in vitro study, a brass tube charged with solder was positioned at the coronal end of the fractured instrument, and Nd: YAP laser irradiation was applied to melt the solder with the intention of connecting the fractured instrument with the brass tube [21]. It is crucial to consider that while removing instruments, the benefits must outweigh the potential damage that may occur during the removal process itself [22]. The excessive removal of dentin may lead to perforation or an increased risk of vertical root fracture [22]. Therefore, an evaluation should be conducted on a case-by-case basis, especially because, to date, there is no gold standard protocol available for managing fractured instruments inside canals [22].

Observing the demonstrated efficacy of a Nd: YAP laser with a 1340 nm wavelength metal cutting stimulated the conceptualization of employing this laser to deal with the difficulty of removing broken files from root canals. Indeed, the capability of a Nd: YAP laser to cut metals represents a significant advantage in this context. It has the potential to facilitate the precise and controlled removal of fractured instruments [23]. The laser’s focused energy allows for the selective ablation of the broken file, reducing the risk of damage to the remaining tooth structure [23,24]. The laser’s high level of control might enable clinicians to limit the removal to the specific area of concern, minimizing the impact on adjacent healthy structures [23,24,25]. Moreover, the flexibility of the tip of the Nd: YAP laser can potentially navigate the intricate and delicate root canal anatomy more effectively. It is interesting to investigate its potential for removing broken files in root canals. However, prior to any clinical application of a Nd: YAP laser for removing fractured Ni-Ti instruments, it is imperative to verify its safety regarding temperature increase during irradiation, potential damage to periodontal tissue, and any potential morphological dentinal damage. Eriksson. et al. [26] suggested in a classic study that the threshold of increase in temperature is 10 °C; hence, irradiation with a Nd: YAP laser should be made in a way to avoid an increase in temperature above 10 °C. Therefore, the objective of this ex vivo study is to establish safe irradiation conditions for a clinical procedure enabling the use of a Nd: YAP laser for removing or bypassing broken nickel–titanium files in root canals. The null hypothesis suggests that there is no difference in safety among different irradiation protocols.

## 2. Materials and Methods

### 2.1. Study Designs

**Figure 1:**
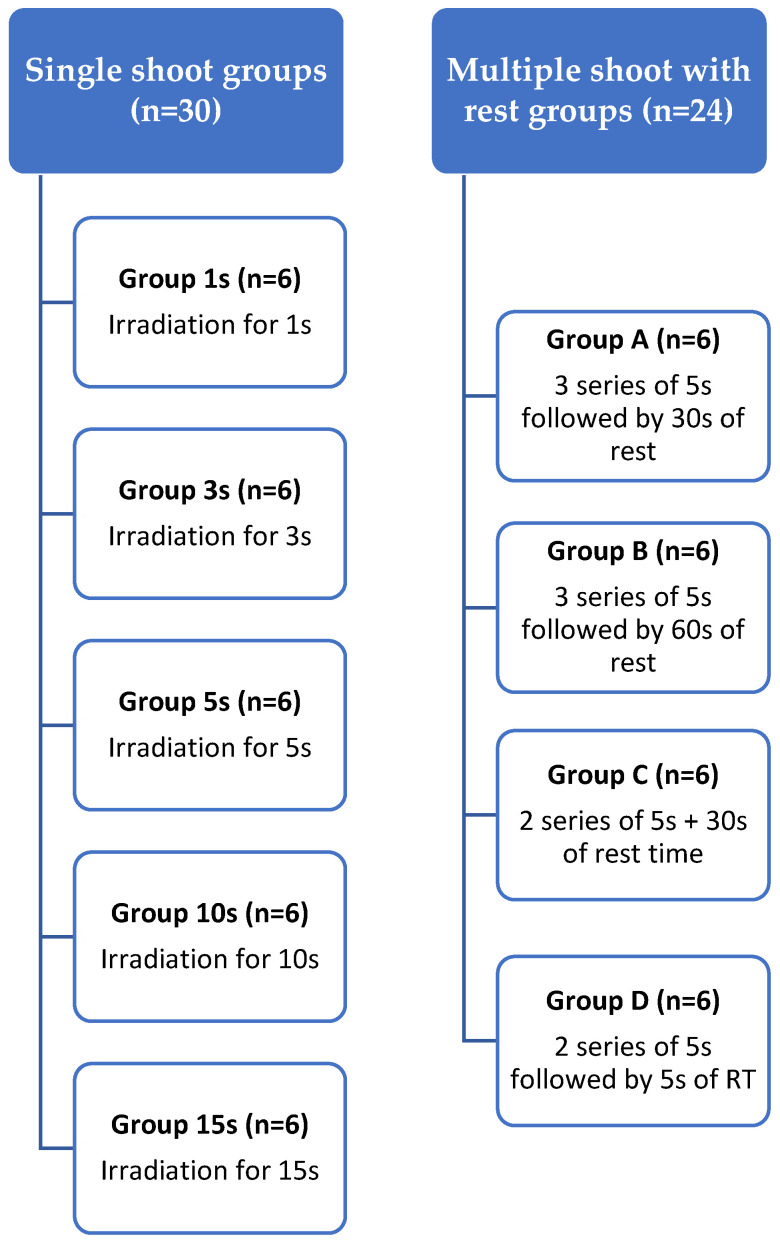
Study design illustrating the nine different groups in this study.

This study aimed to evaluate the safety of the Nd: YAP laser (λ1340 nm, LOBEL MEDICAL SAS, Les Roches de Condrieu, France) treatment protocol in removing broken instruments from permanent tooth roots. The maximum permissible temperature increase was set at 10 °C. This choice was informed by studies conducted by Eriksson and Albrektsson [22], which demonstrated that temperature rises exceeding 10 °C can lead to detrimental effects on the periodontium. Moreover, a SEM assessment was made for all of the specimens in all groups to assess any morphological damage to the irradiated dentinal walls. A total of 54 permanent tooth roots were included (*n* = 54) and divided into 9 groups. Each group had a different irradiation protocol; groups 1 s, 3 s, 5 s, 10 s, and 15 s had an irradiation of one single shoot (Figure 1), while groups A, B, C, and D had a more complex irradiation protocol (explained in Section 2.3) (Figure 1). Ethical committee approval from the University of Liege was not deemed necessary for this study as all included teeth were extracted for reasons unrelated to this research. All patients provided consent for the authors to utilize their extracted teeth for ex vivo research.

### 2.2. Tooth Preparation

A total of 54 permanent human teeth (*n* = 54) were included. All teeth were extracted and collected for reasons that are not related to this study. After collection, the coronal portion of each sample was removed using a fine diamond bur (Meisinger, Neuss, Germany) with continuous water-cooling. The specimens were then washed, cleaned with a scaler (Satelec, Acteon, Norwich, UK), and stored in 0.1% thymol solution at a temperature of four degrees Celsius to prevent microbial growth. Manual endodontic preparation was performed using a standardized step-back serial technique with ISO K-File 10, K-File 15, and K-File 20 (VDW, GmbH, Munich, Germany) until reaching the working length (WL) of the respective roots. Throughout the preparations, continuous irrigation with 3% sodium hypochlorite (NaOCl) was employed.

### 2.3. Procedure for Instrument Fractures

**Figure 2:**
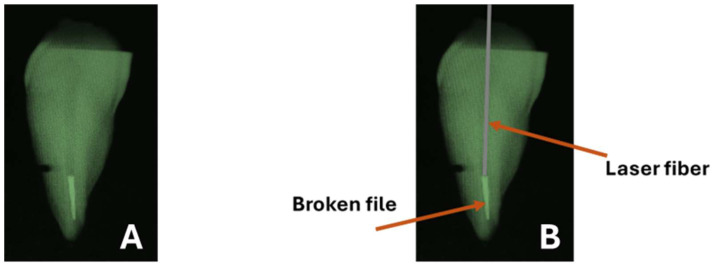
(**A**) A parallel peri-apical X-ray made to confirm the presence of a fractured file at the apical third of the root canal. (**B**) shows the laser fiber positioned in close contact with the broken file.

In order to provoke instrument fracture inside the root canals, the apical 2 mm of R-25 Reciproc files (RECIPROC^®^ R25 File- VDW GmbH) was measured using a graduated ruler and notched with a fine bur until ¾ of the diameter of the file thickness was reached in order to facilitate file separation at the apical part of the root canals. The files were introduced in rotary continuous motion in the root canals and separated with a short bending gesture until the fragment was lodged. The canal obstruction was checked with a 10 K-file (VDW GmbH). If the bypass was possible, the specimens were excluded. To confirm the successful breakage of the instrument at the apical third of the root, parallel radiographs were taken (Figure 2).

### 2.4. Study Groups and Protocol

As already mentioned, 9 groups were included in total in which 5 groups had a single irradiation during variable times: group 1 s (*n* = 6) consisted simply of one second of irradiation, group 3 s underwent irradiation for 3 s (*n* = 6), group 5 s underwent irradiation for 5 s (*n* = 6), group 10 s underwent irradiation for 10 s (*n* = 6), and group 15 s underwent irradiation for 15 s (*n* = 6). Following the identification of the longest and safest duration, additional groups labeled A, B, C, and D were also added to the study.

Group A was composed of 3 series of irradiations of 5 s each separated by a rest time of 30 s (L5s + 30 s RT) (*n* = 6). Group B consisted of 3 series of irradiations of 5 s each separated by a rest time of 60 s (L5s + 60 s RT) (*n* = 6). Group C consisted of 2 series of irradiations of 5 s each separated by a rest time of 30 s (L5s + 30 s RT) (*n* = 6), and group D consisted of 2 series of irradiations of 5 s each separated by a rest time of 5 s (L5s + 5 s RT) (*n* = 6). In all groups, continuous irrigation with 2.5 mL of sodium hypochlorite (3% NaOCl) was carried out during the rest time.

### 2.5. Protocol and Irradiation Parameters

Irradiation with the Nd: YAP laser was initiated when a sensation of a fiber contact with the broken Ni-Ti file was felt inside the root canal. Then, for each group, the irradiation protocol was made according to the description in Section 2.4. Additionally, the laser fiber was equipped with a rubber stopper to indicate to the operator the working length in each root canal. Irradiation parameters for all groups were the same and were as follows: an output power of 3 W, 300 mJ, 955.41 J/cm^2^, a frequency of 10 Hz with a 150 µs/pulse, a fiber diameter of 200 microns, and contact mode.

### 2.6. Assessment of Temperature Increase

A special thermo-conductor paste with a thermal conductivity of 0.4 cal s^−1^ m^−1^ K^−1^ (Warme Leitpaste WPN 10; Austerlitz Electronic, Nuremberg, Germany) was spread on each root surface with the aim of mimicking the thermal conductivity of soft tissues (0.2^−0.5^ cal s^−1^ m^−1^ K^−1^) depending on hydration [24]. The thermo-conductor paste was used to ensure optimal contact and maximal thermal conduction between the sensor tip of the thermocouple probe and the root surface. Each root was closely rounded by one sensor of the probes of the thermocouple located at 2 mm from the apex, and the second sensor was placed into a warm bath in order to control the constancy of the water temperature at 37 °C. A K-type thermocouple (K-type thermocouples HH806AWE Omega, Manchester, UK) with a precision of 0.01 °C was used. Each root was immersed in a 37 °C water bath while keeping the cervical area above the waterline so as to keep water out of the canal. The stability of the temperature at 37 °C was verified. Calculation of the temperature was made by subtracting the Δ of the highest recorded temperature at the root surface from that recorded at baseline (37°). Our protocol for assessing temperature increase and ensuring safety was developed based on established methodologies outlined in previous studies in the literature, specifically the study by Namour et al. [27,28].

### 2.7. Utilization of High-Speed Imaging

To verify the capacity of the Nd: YAP laser to cut endodontic files and to illustrate the interaction between Nd: YAP laser irradiation and a nickel–titanium file, a high-speed imaging system was used, which captured images using high-speed imaging at 3800 frames per second (LaVision, HighSpeedStar, GmbH, Göttingen, Germany) to show the interaction between the Ni-Ti and the activated tip of the Nd: YAP laser during irradiation. This setup allowed for precise observation and analysis of the dynamic process as the laser interacted with the file, providing valuable insights into the mechanism of action and effects of irradiation.

### 2.8. Scanning Electron Microscopy (SEM)

SEM (JSM 7500F, JEOL, Tokyo, Japan) analyses were made to assess any physical changes in the dentinal walls caused by the irradiation protocol. The specimens were dehydrated in blue silicon (with humidity indicator) at room temperature. At that point, they were attached to aluminum stubs and metallized with a layer of gold (25 nm thick) using vacuum evaporation in a metallizer (model SCD 005, Bautec, Berlin, Germany). The samples were then observed under different magnifications.

### 2.9. Statistical Analysis

Statistical analyses were achieved using Prism 5^®^ software (GraphPad Software, Inc., San Diego, CA, USA) to calculate the mean and standard variation values of temperature increase measurements. *p* < 0.05 was considered statistically significant. Confidence level of the study was proposed to be 99% with *p* < 0.001, which is highly significant. Descriptive statistics, including the means and standard deviations, were calculated. ANOVA tests coupled with the Newman–Keuls multiple comparison test (post hoc test) were used.

## 3. Results

### 3.1. Observation from High-Speed Imaging System

**Figure 3:**
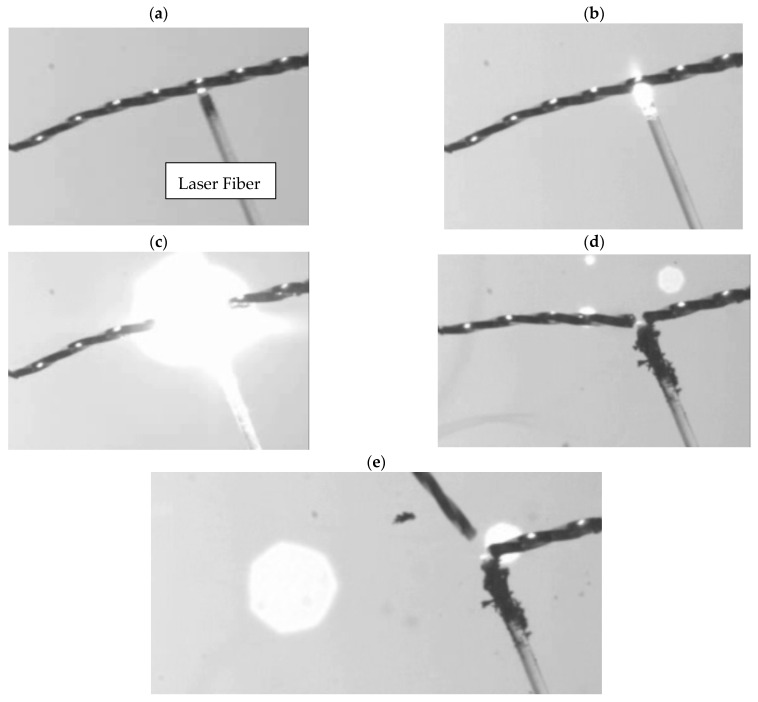
The results of the interaction between Nd: YAP laser irradiation and a nickel–titanium file, captured using high-speed imaging at 3800 frames per second. (**a**) The laser tip in contact with the Ni-Ti file before irradiation. (**b**) At the beginning of the irradiation. (**c**) During irradiation. (**d**) The Ni-Ti instrument breaks immediately after irradiation. (**e**) The division of the two components of the Ni-Ti file.

The analysis of the trial involving the cutting of a Reciproc R25 File using a Nd: YAP laser, conducted with a high-speed camera, demonstrated the laser’s effectiveness in cutting the file within 1 s of laser firing (Figure 3).

### 3.2. The Results of the Temperature Increase in the Single Irradiation Groups

**Figure 4:**
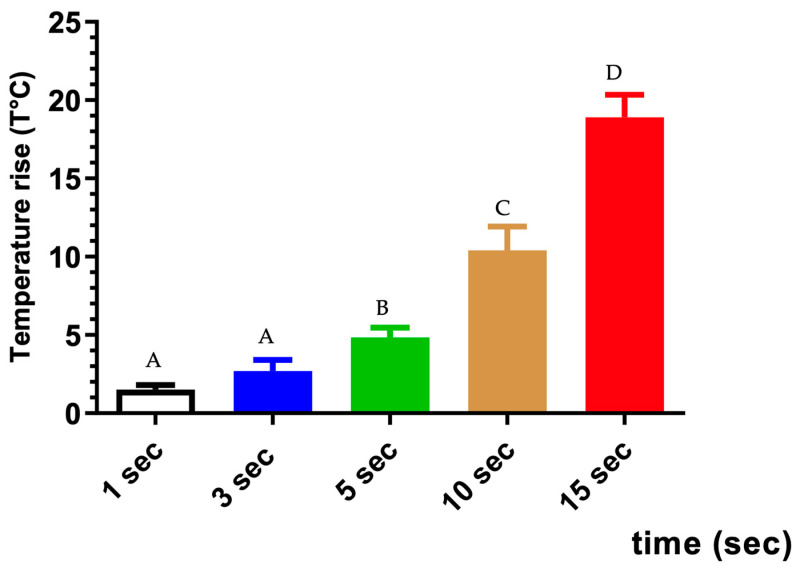
The mean and standard deviations of the temperature increase in the groups with a single shoot group (1 s, 3 s, 5 s, 10 s, and 15 s). Identical superscript letters indicate the absence of a statistically significant difference. The difference in superscript letters indicates a statistically significant difference. *p*-value < 0.0001.

All samples passed the normality test, indicating a Gaussian distribution of values. The mean values of ΔT increased with longer irradiation times as follows: group 1 s < group 3 s < group 5 s < group 10 s < group 15 s. Only groups 10 s and 15 s exceeded the maximum temperature threshold, with values of 10.40 ± 1.52 °C and 18.93 ± 1.43 °C, respectively. In contrast, the mean ΔT for the other groups remained within acceptable limits, with values of 1.51 ± 0.33 °C, 2.73 ± 0.70 °C, and 4.83 ± 0.62 °C for groups 1 s, 3 s, and 5 s, respectively (refer to Table 1 and Figure 4).

### 3.3. Results of Temperature Increase in Groups with Multiple Irradiation and Rest Time

**Figure 5:**
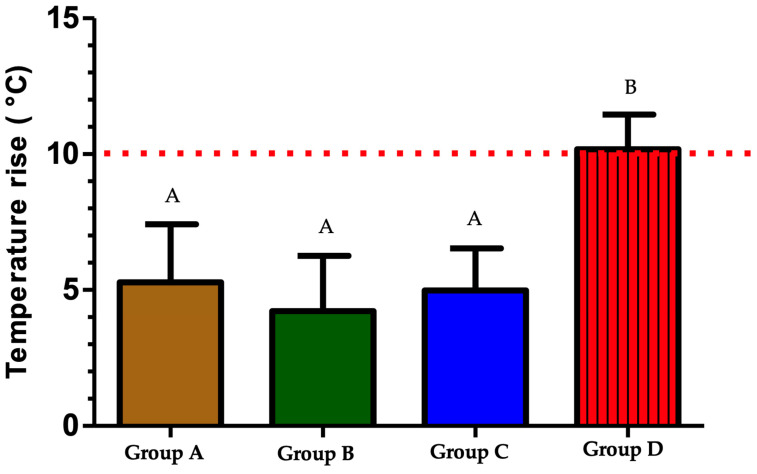
The results of the temperature increase in different groups. Identical superscript letters indicate the absence of a statistically significant difference. The difference in superscript letters indicates a statistically significant difference. *p*-value < 0.0001.

All samples passed the normality test, indicating a Gaussian distribution of values. Among the groups, group D exhibited the highest mean value and standard deviation, measuring 10.18 ± 0.4252 °C. This was statistically significantly higher compared to groups C, B, and A, which recorded values of 4.983 °C, 4.224 °C, and 5.282 °C, respectively. Furthermore, there was no statistically significant difference observed between groups A, B, and C. All of these groups demonstrated an increase in temperature below the predefined threshold of 10 °C. Therefore, except for group D, all groups were deemed safe for periodontal tissue, as their mean temperature increase remained below the threshold (<10 °C) (Figure 5, Table 2). The null hypothesis was rejected.

### 3.4. The Results of the SEM Analysis

**Figure 6:**
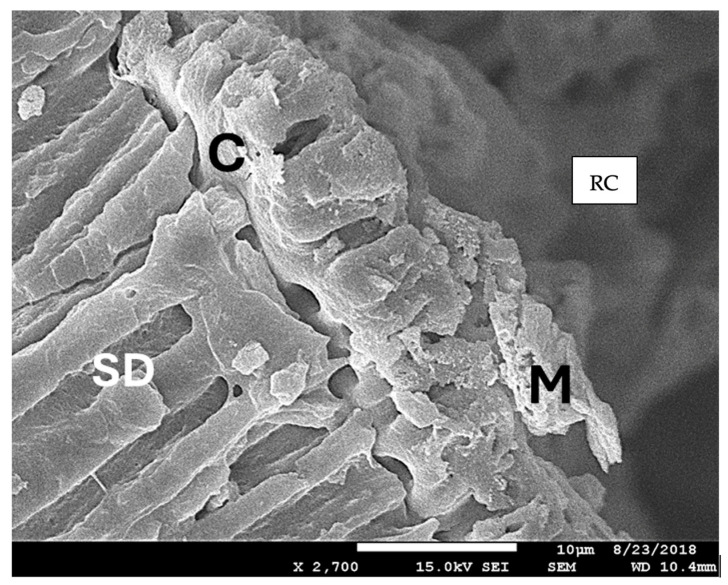
A scanning electron microscopy image at 2700 × magnification illustrating dentinal melting (M) and tubule closure on the canal walls at the site of the complete removal of the fractured file. The healthy dentin located below the thermally affected zone exhibits a normal structure (SD). However, a distinct crack (C) separating zones M and SD is observed. The irradiated root was fractured longitudinally and observed with SEM. RC shows the root canal area at the level of the removed instrument.

The SEM analysis revealed that rare and limited microcracks were present across all groups. However, the dentinal walls exhibited signs of dentinal melting and tubule closure, which were attributed to the removal of the fractured file (Figure 6). Additionally, a crack was noted, separating the melted surface zone from the underlying healthy dentin (Figure 6).

## 4. Discussion

The fracture of endodontic instruments within the root canal (RC) system, commonly referred to as instrument separation, is an iatrogenic incident that warrants diligent efforts to minimize its occurrence [29]. Effective management becomes crucial in the event of such an occurrence, particularly as it impacts the processes of cleaning, rinsing, shaping, and filling the root canal. Any alteration in these procedures can lead to a significant decrease in the overall success rate of the treatment [30,31]. As mentioned in the introduction, presently, there is no universally accepted protocol for managing instrument separation within an RC [22]. Nevertheless, the complete removal or bypass of the fractured instrument is widely regarded as a successful approach in addressing this iatrogenic complication [22]. However, a thorough analysis of the case is imperative prior to the removal of a broken instrument. The advantages must outweigh the potential risks that may arise during the removal procedure [22]. Excessive dentin removal can result in perforation or a heightened susceptibility to vertical root fracture. Hence, an assessment should be performed on an individual basis, particularly since, as of now, there is not a universally accepted protocol for addressing fractured instruments within root canals [22].

Prior to proposing a protocol for removing broken instruments, its safety and effectiveness must be analyzed and evaluated. Safety considerations should encompass the potential increase in temperature during the protocol and the likelihood of morphological damage, both of which merit a thorough investigation [26,27,28]. Hence, in this study, an SEM analysis was conducted to evaluate any physical alterations in the dentinal walls due to laser irradiation. Additionally, a specialized thermo-conductor was employed to monitor the temperature variations across different protocols (different irradiation times), ensuring that they remained within the acceptable threshold. This comprehensive approach not only validated the Nd:YAP laser’s cutting capacity but also provided insights into its impact on endodontic materials, which are crucial for optimizing clinical applications while ensuring patient safety.

For instance, an increase in temperature above the threshold might result in irreversible side effects on the periodontium, such as hyperemia, the darkening and resorption of fat cells, followed by a fat cell invasion, increased capillary leakage, and thermally induced bone necrosis [26]. In this matter, Erkisson [26] et al. showed in a histologic study that bone tissue heated to 47 °C can remain as functioning bone but will become resorbed and replaced with fat cells, and extreme cases of heating to 47 °C for 1 min causes a fat cell injury but an inconsistent bone injury [26]. Hence, the aim of this study was to evaluate the safety of the suggested protocol and to propose the most effective and safe irradiation protocol and parameters. To achieve this, thermo-conductors, scanning electron microscopy, and high-speed imaging systems were used in this study. After demonstrating the safety of the protocol, further studies focusing on its effectiveness can be conducted.

Regarding the safety of using a Nd: YAP laser, this ex vivo study revealed that its safety can vary depending on the irradiation protocol and parameters. This was clearly demonstrated in the study results. For example, when used with two sets of 5 s irradiation periods, each followed by 5 s of rest time, or when used continuously for 10 s, an increase in temperature above 10 °C was observed, indicating that harm was caused to the periodontium. Another finding of this study was the effectiveness of the Nd: YAP laser to cut endodontic instruments, as demonstrated by high-speed imaging at 3800 frames per second and an SEM analysis. This imaging system revealed that, within seconds, the complete cutting of the Ni-Ti instrument occurred upon irradiation. Additionally, a SEM analysis indicated the melting of the contacted dentin following irradiation with the Nd: YAP laser.

The Nd: YAP laser was selected for its specific characteristics that could be advantageously utilized for removing broken Ni-Ti instruments from the root canal system. The selection of this laser wavelength (1340 nm) was based on its absorption properties by metals, including Ni-Ti falling within this near-infrared range [23,24,25,27]. This wavelength allows for significantly efficient energy absorption by metals, which was considered an advantage for our main objective of precisely targeting and manipulating the broken instrument within the complex root canal system [23,24,25]. This controlled interaction with the broken instrument might minimize potential damage to the surrounding tissues, which could theoretically lead to the successful removal of the broken instruments; hence, it is a promising solution.

In the literature, the utilization of a Nd: YAP laser as an adjunctive method for enhancing canal cleaning in endodontic and restorative dentistry, as well as its antimicrobial efficacy, has been documented [32,33,34]. The bactericidal effect of the Nd: YAP laser primarily relies on elevating the temperature within the root canal, leading to bacterial eradication [35,36,37]. However, its application for the extraction of fractured instruments lacks comprehensive exploration [35,36,37]. Farge et al. [37] reported that the combination of a Nd: YAP laser and conventional instrumentation presents an advantage in endodontic re-treatments in terms of removing canal sealers and broken instruments [37]. In one study, a Nd: YAP laser was able to destroy the sealer by staying in contact with it without affecting the dentinal walls [37]. These authors also portrayed that using hand instrumentation after irradiation is necessary to evacuate the carbonized cement and to enlarge the canal wall due to the accumulation of carbonized dentinal or sealer debris, which can lead to apical plug [37]. Along with that, the study concluded that using 200 mJ with 10 Hz did not result in morphological damage to the visible dentin [37]. A similar indication, albeit with a Nd: YAG laser, was found in the literature. Gruber et al. [21] proposed a protocol for the removal of broken instruments from the root canal using a Nd: YAG laser [21]. The protocol consisted of creating a fixed connection between the fractured instrument and a solder inside a small brass tube and then melting both the fractured instrument and the solder with the Nd: YAG laser [21].

The conclusion drawn from our study suggests that the selected protocol may be safe in terms of temperature increase and morphological changes during irradiation for 5 s. This paves the way for the development of a novel approach that consists of using a Nd: YAP laser with these parameters for irradiation and protocols.

Hence, future ex vivo investigations can evaluate the effectiveness of removing broken Ni-Ti instruments in the root canal using our selected laser-assisted Nd: YAP irradiation protocol.

## 5. Conclusions

This study unveiled that the safest protocol for bypassing and/or removing broken instruments involves three series of irradiations 5 s each separated by a 30 s rest period. This recommended protocol can be considered safe in terms of temperature increase for periodontal tissues and morphological changes in dentinal tissues. Furthermore, it was found that irradiation lasting continuously for 10 s or more could pose a risk of harm to periodontal tissue.

## Figures and Tables

**Table 1:** Mean and standard deviations of temperature increase in groups with single shoot group (1 s, 3 s, and 5 s).

	Groups				
	1 s (*n* = 6)	3 s (*n* = 6)	5 s (*n* = 6)	10 s (*n* = 6)	15 s (*n* = 6)
Mean ∆T (°C)	1.51 ^A^	2.73 ^A^	4.83 ^B^	10.40 ^C^	18.93 ^D^
Standard deviations	0.30	0.70	0.62	1.52	1.43
Below the limit for temperature rise	YES	YES	YES	NO	NO

Identical superscript letters indicate the absence of a statistically significant difference. The difference in superscript letters indicates a statistically significant difference. *p* value < 0.0001.

**Table 2:** Means and standard deviations of temperature increases during broken file removal using Nd: YAP laser during different experimentations.

Groups	Description	Mean ΔT (°C)	Std. Deviation (°C)	Std. Error (°C)	Below The Limit for Temperature Rise
A	3 sets of (L5s + 30 s RT)	5.282 ^A^	2.136	0.5181	YES
B	3 sets of (L5s + 60 s RT)	4.224 ^A^	2.027	0.4423	YES
C	2 sets of (L5s + 30 s RT)	4.983 ^A^	1.542	0.6295	YES
D	2 sets of (L5s + 5 s RT)	10.18 ^B^	1.276	0.4252	NO

Identical superscript letters indicate the absence of a statistically significant difference. The difference in superscript letters indicates a statistically significant difference. *p*-value < 0.0001. L = laser irradiation; s = seconds; RT = rest time.

## Data Availability

The data are available upon reasonable request.
